# Auscultation and Percussion

**Published:** 1854-12

**Authors:** 


For sale by Miller, Orton & Mulligan. H.

---

ART. VII.—*Auscultation and Percussion.* By Dr. Joseph Skoda. Translated from the Fourth Edition, by W. O. Markham, M. D., Assistant Physician to St. Mary's Hospital. Philadelphia: Lindsay & Blakiston. 1854.

The doctrines of Skoda have been largely discussed by various critics, some of whom have not helped us very essentially to a clear understanding of his distinctive doctrines. The translator has given us a preface, which saves the trouble of writing a review, by giving us a clear view of Skoda's peculiar doctrines on the broncophonic voice, which we accordingly present for the consideration of our readers, confident that we are doing better justice to the book than by anything we could offer in the way of comment.

"* * * The voice passes into the parenchyma of the lungs through the medium of the air in the trachea and the bronchial tubes, and is not propagated along their walls; it traverses healthy, as readily as it does hepatized lungs, and even somewhat more readily: consequently, bronchophony does not depend upon an increase of the sound-conducting power of consolidated pulmonary tissue; moreover, when the lung is consolidated, the thoracic voice increases and diminishes in force, without any concurrent change taking place in the condition of the lung; this variation in its strength evidently results from the circumstance of the bronchial tubes being at one moment blocked up by mucus, etc., and at another freed therefrom by the cough and expectoration, etc.: if the bronchophony depended upon conduction of sound, it would be a matter of indifference whether the tubes contained air or fluids. It must not be forgotten, that, according to the ordinary laws of reflection of sound, the more solid the parenchyma the more difficult does the passage of the sound from the air into it become.

"That the air in the mouth and nasal cavities consonates with sounds formed in the larynx, is proved by the fact of the changes which the voice undergoes through opening and closing the mouth and nose, whilst the condition of the larynx remains unaltered; just in the same way does the air in the trachea and bronchial tubes consonate with the laryngeal sounds. Now, air consonates only in a confined space, and the force of the consonance depends upon the form and size of the space, and upon the nature of the walls forming it: the more solid the walls, the more completely will the sound be reflected, and the more forcible the consonance. The cause of the loud voice produced by a speaking-trumpet is well-known. But the air will consonate with certain sounds only; in the trachea and bronchial tubes, it becomes consonant with the laryngeal voice, in so far as their walls have a like or an analogous character to the walls of the larynx, of the mouth, and of the nose. Within the cartilaginous walls of the trachea and the bronchial trunks, the voice consonates nearly as forcibly as in the larynx; but as the bronchial tubes divide in the lungs, they lose their cartilaginous character, becoming at least merely membraneous in structure, and therefore, very ill-adapted for consonance; when, therefore, the consonance is increased in these latter tubes, we may be sure, either that the membrane forming them has become very dense or cartilaginous, or that the tissue around them is condensed and deprived of air, whereby the sound-reflecting power of the tubes is increased. Of course the communication between the air in the tubes and the air in the larynx must be uninterrupted.

"The walls of a confined space frequently vibrate in unison with sounds excited within it, as do those of an organ-pipe, or of a speaking-trumpet. The larynx vibrates with every sound, and its vibrations are perceptible at a considerable distance from their point of origin; so, also, must the walls of the bronchial tubes, which are distributed through the parenchyma of the lungs, vibrate when the voice consonates within them; and the vibrations thus excited will extend to the surface of the thorax, passing through several inches of thick, fleshy parts, or of fluids, and manifest themselves there as the consonating sounds of the bronchial tubes. * * *"

